# Development of Hydrogen–Oxygen Fuel Cells Based on Anion-Exchange Electrolytes and Catalysts with Reduced Platinum Content

**DOI:** 10.3390/membranes13070669

**Published:** 2023-07-14

**Authors:** Oleg Korchagin, Vera Bogdanovskaya, Inna Vernigor, Marina Radina, Irina Stenina, Andrey Yaroslavtsev

**Affiliations:** 1 Frumkin Institute of Physical Chemistry and Electrochemistry, Russian Academy of Sciences, 119071 Moscow, Russia; oleg-kor83@mail.ru (O.K.); msnoviinna@gmail.com (I.V.); merenkovamarina@mail.ru (M.R.); 2 Kurnakov Institute of General and Inorganic Chemistry, Russian Academy of Sciences, 119071 Moscow, Russia; stenina@igic.ras.ru

**Keywords:** hydrogen–oxygen FC, anion-exchange membranes, architecture of membrane–electrode assembly, non-platinum catalytic system, anodic catalysts with reduced platinum content

## Abstract

Studies have been carried out to optimize the composition, formation technique and test conditions of membrane electrode assemblies (MEA) of hydrogen–oxygen anion-exchange membranes fuel cells (AEMFC), based on Fumatech anion-exchange membranes. A non-platinum catalytic system based on nitrogen-doped CNT (CNT_N_) was used in the cathode. PtMo/CNT_N_ catalysts with a reduced content of platinum (10–12 wt.% Pt) were compared with 10 and 60 wt.% Pt/CNT_N_ at the anode. According to the results of studies under model conditions, it was found that the PtMo/CNT_N_ catalyst is significantly superior to the 10 and 60 wt.% Pt/CNT_N_ catalyst in terms of activity in the hydrogen oxidation reaction based on the mass of platinum. The addition of the Fumion ionomer results in minor changes in the electrochemically active surface area and activity in the hydrogen oxidation reaction for each of the catalysts. In this case, the introduction of ionomer–Fumion leads to a partial blocking of the outer surface and the micropore surface, which is most pronounced in the case of the 60Pt/CNT_N_ catalyst. This effect can cause a decrease in the characteristics of MEA AEMFC upon passing from 10PtMo/CNT_N_ to 60Pt/CNT_N_ in the anode active layer. The maximum power density of the optimized MEA based on 10PtMo/CNT_N_ was 62 mW cm^−2^, which exceeds the literature data obtained under similar test conditions for MEA based on platinum cathode and anode catalysts and Fumatech membranes (41 mW cm^−2^). A new result of this work is the study of the effect of the ionomer (Fumion) on the characteristics of catalysts. It is shown that the synthesized 10PtMo/CNT_N_ catalyst retains high activity in the presence of an ionomer under model conditions and in the MEA based on it.

## 1. Introduction

In recent years, there has been a growing interest in fuel cells (FCs) based on anion-exchange membranes (AEMFCs), since they offer a number of potential advantages over those based on proton-exchange membranes (PEMFCs). One of the main advantages is their higher pH value in the zone of electrochemical reactions, which is approximately 0 for PEMFCs, while for AEMFCs in the OH form, it ranges between 13–14. In an alkaline environment, the overvoltage of the oxygen reduction reaction (ORR), which typically determines the overall voltage drop during FC operation [1,2], is reduced, along with the rate of corrosion of membranes and catalysts in FC. This offers a wider range of materials that can be used in bipolar plates, membranes and catalysts [3]. Due to the aforementioned advantages of AEMFCs, it is possible to use non-platinum catalysts in a membrane–electrode assembly (MEA), which can help to reduce the cost of generated energy [3,4].

By implementing AEMFC technology, it becomes possible to use new polymer electrolytes and catalysts, along with optimizing operating conditions. Unlike PEMFCs, AEMFCs involve the participation of water, formed at the anode, in the oxygen reduction reaction at the cathode, following the reaction schemes:

H_2_ + 2OH^−^ → 2H_2_O + 2ē (1)

1/2O_2_ + H_2_O + 2ē → 2OH^−^ (2)

For the AEMFC-efficient operation, it is important to prevent flooding of an active anode layer and ensure the water transport to a cathode through a polymer membrane. In this regard, achieving a water balance in AEMFC is a more complex task compared to PEMFC [3]. According to the literature [5], the diffusion of water from the anode to the cathode is the primary source of water for the cathode’s reaction. This water diffusion also keeps the membrane hydrated, which helps to maintain its high ionic conductivity and reduce the rate of degradation.

Currently, non-platinum catalysts have mainly been used as cathode catalysts in AEMFC MEAs. Nitrogen-doped carbon materials with or without embedded transition metals in the catalyst structure are among the most studied non-platinum catalysts for ORR [3,6]. By using an Fe/CNT cathode catalyst and a PtRu/C anode catalyst [3], the maximum AEMFC power density of 1.44 W cm^−2^ was achieved, which represents the best result obtained so far for both AEMFC and PEMFC with non-platinum cathode catalysts. The examples of successful practical applications of non-platinum catalysts for AEMFC anodes include the investigation of the Ru_7_Ni_3_/C system for hydrogen oxidation reaction (HOR) [7], as well as the development of HOR catalysts based on palladium modified with nickel and cerium compounds [8,9]. However, the current price of palladium exceeds that of platinum by almost 2.5-fold, and the corrosion resistance of palladium within catalysts is generally inferior to that of platinum [10,11,12]. Therefore, reducing the platinum content is considered as a more promising approach to the development of affordable and stable HOR catalysts, rather than developing systems based on other platinum group metals. Molybdenum attracts the attention of researchers due to its relatively low cost, as well as catalytic activity comparable to PtRu, in HOR [13]. It was shown in [14] that alloying platinum with molybdenum increases both its catalytic activity and stability in the reaction of hydrogen oxidation in fuel cells. The choice of molybdenum as the second metal is due to the fact that it is one of the available elements that act as a “source” of electrons [15].

In the literature describing AEMFCs with high characteristics, typically lab-made polymer membranes and corresponding ionomer solutions were used [1,5,7,12,16]. Commercial anion-exchange polymer electrolyte manufacturers such as Tokuyama (Tokyo, Japan), Sustainion and FuMA-Tech Inc. (Fumatech) (Bietigheim-Bissingen, Germany) offer a range of products [17]. Fumasep electrolytes consist of an aminated poly(phenylene oxide) backbone with quaternary ammonium groups [18] and find use in various applications, including hydrogen production by electrolysis of water [19], electrodialysis [20] and acid regeneration [21]. However, relatively few works have investigated the use of Fumasep membranes in AEMFC MEA [22,23,24,25] and the use of platinum-based cathodic and anodic catalysts when forming MEA.

The addition of an anion-exchange ionomer, a polymer electrolyte solution compatible with a membrane, into an active layer (AL) of an electrode enables the transport of OH ions into the reaction zone. The optimal content of the ionomer in AL depends on its ion-exchange capacity, the mobility of OH groups, and the ability of cations in the ionomer to interact with active centers in the catalyst. The use of anion-conducting polymers with varying compositions and characteristics leads to different optimal ionomer contents ranging from 50 to 150 wt.% relative to the amount of carbon material used as a catalyst carrier [5,22,23,26]. The type and concentration of solvent in the catalytic ink, as well as the AL-forming conditions, significantly influence the structure and characteristics of AL. In the study [24], it was found that incorporating low-boiling solvents such as methanol and tetrahydrofuran into 10 wt.% solution of Fumion ionomer in high-boiling n-methyl pyrrolidone (NMP) allows the AL porosity to be increased, resulting in high AEMFC performance. Another study [22] reported positive results when fabricating FAA ionomer inks using an aqueous solution of isopropyl alcohol.

In this work, studies were carried out in order to optimize the fabrication technique and test conditions of the hydrogen–oxygen AEMFC based on anion-exchange membranes manufactured by Fumatech, namely, Fumasep^®^ membranes and Fumion ionomer. A catalytic system based on carbon nanotubes (CNTs) doped with nitrogen (CNT_N_) was used as a part of the cathode AL, while a PtMo/CNT_N_ catalyst with reduced platinum content (10–12 wt.% Pt) was used at the anode.

## 2. Materials and Methods

### 2.1. Catalysts Synthesis

#### 2.1.1. The Synthesis of the Cathode Catalyst

The synthesis of the cathode CNT_N_ catalyst involves several sequential stages. First, 400 mg of initial CNTs (Nanotechcenter LLC, Tambov, Russia) was placed in a flask under reflux; 200 mL of 1 M NaOH (Chimmed LLC, Moscow, Russia) was added. The mixture was kept for 1 h at 100 °C under continuous stirring using a magnetic stirrer. Further, the functionalized CNTs were washed with deionized water until neutral pH was reached. The resulting precipitate was dried in a vacuum oven at 90 °C until completely dry. In the next stage, CNTs were mixed with melamine (Alfa Aesar, Ward Hill, MA, USA) as a nitrogen precursor at a ratio of 1:0.7, followed by milling in a ball mill for 1 h at 800 rpm. The resulting mixture was placed in a quartz tube and subjected to thermal treatment in an Ar atmosphere at 600 °C for 1 h. The nanotubes obtained following nitrogen doping were designated as CNT_N_.

#### 2.1.2. Synthesis of PtMo/CNT_N_ Anode Catalysts

In order to synthesize the PtMo/CNT_N_ catalyst, ammonium heptamolybdate tetrahydrate (Alfa Aesar, USA) was dissolved in 1 mL of H_2_O and mixed with the required amount of H_2_PtCl_6_ water solution, followed by the addition of 4 µL of formic acid to the CNT suspension. The resulting mixture was evaporated in a water bath under constant stirring. The dry powder was subjected to heat treatment at 440 °C in an Ar atmosphere for 2 h, followed by cooling in argon to room temperature.

#### 2.1.3. Polyol Synthesis of Pt/CNT_N_ Catalyst

CNT_N_ were dispersed in ultrapure ethylene glycol (EKOS-1, Moscow, Russia) using ultrasonic agitation for 1 h. Subsequently, the resulting suspension was transferred to a round-bottom flask immersed in a laboratory bath filled with glycerol. A solution containing H_2_PtCl_6_·6H_2_O (Aurat, Moscow, Russia) was added to the ethylene glycol suspension using a drop funnel. The mixture was heated under argon barbotage at 110–130 °C for 1.5 h. The resulting product was settled, washed with water, and the solid precipitate was separated by centrifugation and placed in a desiccator.

### 2.2. Methods of Electrochemical Characteristics under Model Conditions

The experiments were carried out using a three-electrode electrochemical cell with separate electrode compartments. The carbositall disk electrode (S_geom_ = 0.126 cm^2^) was used as a working electrode, platinum wire acted as an auxiliary electrode and Hg/HgO was used as a reference electrode. The measurements were performed in 0.1 M KOH. All potential values are given relative to the reversible hydrogen electrode (RHE).

To prepare the catalytic ink, 2 mg of the catalyst was dispersed in 500 μL of isopropyl alcohol. Further, 5 μL (~150 μg cm^−2^) of this suspension was deposited onto the surface of the working disk electrode using a micropipette and air-dried at room temperature for approximately 30 min. To evaluate the effect of adding Fumion ionomer, a 10 wt.% solution of Fumion (I) was added to the suspension in an amount equal to the mass of carbon material (C) in the catalyst (I/C = 1/1), similar to the preparation of catalytic layers for electrodes in MEAs.

Cyclic voltammetry (CV) was performed using a stationary electrode in an argon atmosphere with a scan rate of 0.05 V s^−1^. The electrochemically active surface area (S_EAS_) of Pt was determined by integrating the charge in the I–V curves associated with hydrogen desorption, assuming that 0.210 μC cm^−2^ is required for a monolayer of hydrogen to fully occupy 1 cm^2^ of the Pt surface.

To evaluate the activity of the investigated materials in the HOR, polarization curves were measured in an electrolyte saturated with H_2_. The scan rate was 0.005 V s^−1^ at a rotation speed of the electrode of 1500 rpm. The catalytic activity was determined by the overvoltage values at a current density of 1.5 mA cm^−2^. Current transients were recorded on a rotating disk electrode (650 rpm) at a potential of 0.4 V.

### 2.3. Methods for Studying the Structure of Catalysts

#### 2.3.1. Brunauer–Emmett–Teller (BET) Method

The porous structure of the catalyst samples (60Pt/CNT_N_, 10 PtMo/CNT_N_, and the same samples with the addition of Fumion in the same amount as used for the electrochemical measurements) was examined by low-temperature nitrogen adsorption using an automatic surface area and porosity analyzer, Gemini VII (Micromeritics, Norcross, GA, USA). The obtained adsorption isotherms for all materials belonged to the type IV isotherm according to the BET method, which is characterized by polymolecular adsorption followed by capillary condensation in mesopores. The shape of the hysteresis loop corresponds to the slot shape of the mesopores. By using the experimental data, the main characteristics of a porous structure of each material were calculated, including specific surface area, pore size distribution and pore volume. Pores with a diameter of less than 2 nm were classified as micropores, and those with a diameter higher than 2 nm were classified as mesopores.

#### 2.3.2. X-ray Photoelectron Spectra (XPS) and Scanning Electron Microscopy

XPS spectra were obtained using an Auger spectrometer (Vacuum Generators, St Leonards-on-Sea, UK) equipped with a CLAM2 attachment for XPS measurements. The analyzer chamber was evacuated to a pressure of 10^−8^ Torr. Monochromatic radiation was generated by an X-ray tube with an aluminum anode at a power of 200 W. The peak positions were referenced to the C1s peak at an energy of 285.0 eV. Quantitative ratios were determined using sensitivity coefficients provided in the VG1000 software for processing spectra. The surface composition was determined to a depth of 10 nm.

The morphology of the obtained catalysts was studied using a scanning electron microscope (SEM) (Tescan Amber (Brno, Czech Republic)) equipped with the AZtec (Oxford Instruments, Abingdon, UK) system for energy-dispersive X-ray spectroscopy.

### 2.4. Spectrophotometric Measurements

Platinum content in the catalysts was determined by measuring the optical density of the solution of the Pt complex with bivalent tin chloride (SnCl_2_) over a wide concentration range. The optical density of the solution was measured on a SPECORD M40 spectrophotometer. A more detailed measurement procedure can be found in the reference [27].

### 2.5. Methodology for Forming and Testing AEMFC-Based MEAs

**Figure 1:**
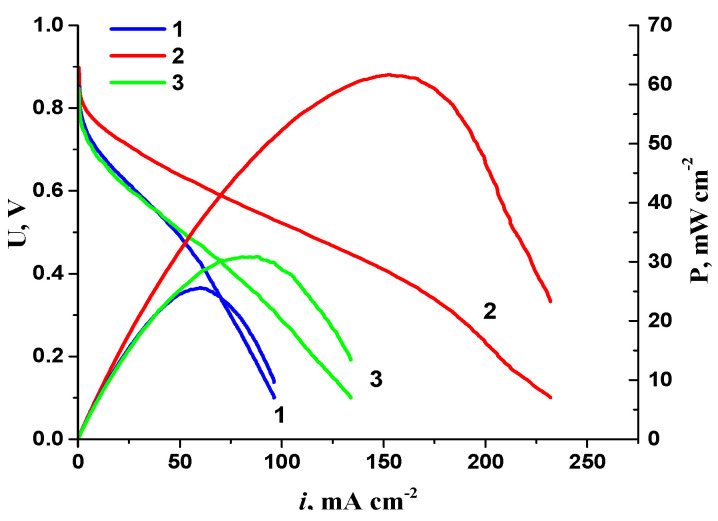
I–V curves and dependence of power density on current density for AEMFC MEA when the membrane and electrodes were kept in 1 M KOH for 2 h (1), 4 h (2) and 4 h with a four-fold replacement of a solution (3).

50 μm-thick Fumasep anion-exchange membranes were used to create MEAs: FAA-1 (first generation), FAS-50 and FAA-3-50, as well as a solution of Fumion ionomer (in Br form) in NMP (FuMA-Tech Inc., 10 wt.%). The electrodes were formed by spraying a suspension of ionomer and catalysts with an aqueous solution of isopropanol, having an isopropanol–to–water ratio of 3:2 (according to the data [26]), unless otherwise specified, on a gas diffusion layer (GDL), type Sigracet 39 BB (thickness 280 µm, fluoroplastic content 5%). The ionomer–to–carbon carrier (I/C) ratio in the active layers was typically 1:1. In specific experiments, the impact of adding other solvents (tetrahydrofuran (THF), NMP) to the suspension, using GDL type 10 CC (350 μm thick, 10% fluoroplastic content), and varying the I/C ratio on MEA AEMFC characteristics was investigated. On the basis of previously obtained results [28], the CNT_N_ catalyst content in the cathodic active layer was selected (1 mg cm^−2^). The anode AL was loaded with approximately 0.20 mg_Pt_ cm^−2^ of 10PtMo/CNT_N_ catalyst. Prior to testing, the membrane and electrodes were immersed in 1 M KOH solution for 4 h. The solution volume corresponded to an excess of OH groups of over 100 times the ionogenic group content in the membrane and electrodes (1.6–2 mmol/g according to the technical specification). Preliminary experiments showed that using a high concentration of alkali (2 M KOH), reducing the exposure time to 2 h, or increasing it to 24 h led to no positive results. Despite the results of a previous study [22], repeated replacement of the alkaline solution (1 M KOH) also failed to yield highly reproducible MEA parameters (Figure 1).

Following exposure, the membrane was placed between the electrodes in a 1 cm^2^ ElectroChem MEA test cell. The degree of compression of the MEA was adjusted by selecting the thickness of the Teflon spacers. In contrast to the technique used for forming MEAs based on Nafion polymer electrolytes, where a compression ratio of 20–25% relative to the initial thickness is typically used [29], a reduced compression ratio (~10%) was found to be more effective for MEAs with Fumasep membranes.

The AEMFC MEA was tested using an ElectroChem test bench. Oxygen was supplied to the cathode space of the cell, while hydrogen was supplied to the anode space at a gas flow rate of 300 mL/min. The relative humidity of the gases was maintained at 100% unless specified otherwise. The cell temperature was set to the maximum allowable temperature of Fumasep membranes according to the producer (40 °C). Electrochemical measurements were carried out using an Elins P-45x potentiostat–galvanostat, equipped with an FRA module.

Upon achieving a steady open-circuit voltage (OCV) between 0.8 to 1 V, the MEA was activated by the expose at 0.1 V [23]. During activation, oxygen (2) was reduced to hydroxyl ions at the cathode, which were further transferred from the cathode to the anode, removing impurities (mainly carbonate anions) from the membrane. This led to an increase in the pH and ionic conductivity of the electrolyte [30]. The increase in ionic conductivity was further enhanced by the formation of water due to the anodic reaction (1), which moistened the membrane. The activation process was terminated once the current increase ceased. Subsequently, the MEA was tested by recording quasi-stationary I–V curves (voltage scan rate of 5–10 mV/s) from OCV to 0.1 V. Once reproducible current–voltage characteristics were obtained, an electrochemical impedance spectrum was measured in the frequency range of 1 Hz to 50 kHz from the OCV with an amplitude of 10 mV. The high-frequency resistance of the MEA (R^HF^) was determined by the point of intersection of the Nyquist plot with the axis of actual resistance values.

## 3. Results and Discussion

**Figure 2:**
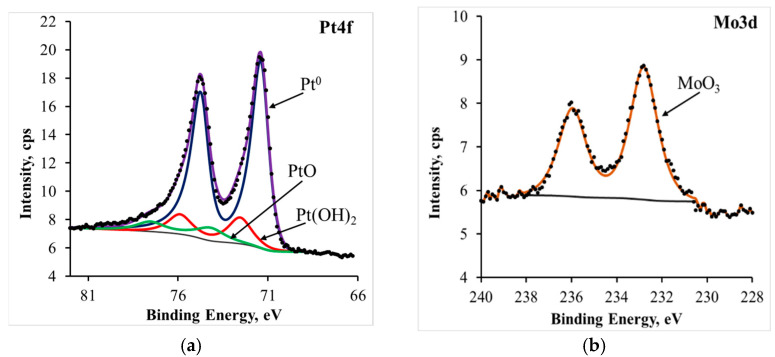
XPS spectra of a catalyst 10PtMo/CNT_N_: Pt4f (**a**) and Mo3d (**b**).

Based on XPS data, the 10PtMo/CNT_N_ catalyst predominantly contains platinum in the metallic state, as well as in the form of an oxide (Figure 2). Conversely, molybdenum was found only in the oxidized state as MoO_3_, which can be attributed to its highly negative redox potential, which is consistent with previous findings [31]. The surface concentration of metal amounted to 0.36% (Pt) and 0.16% (Mo).

**Figure 3:**
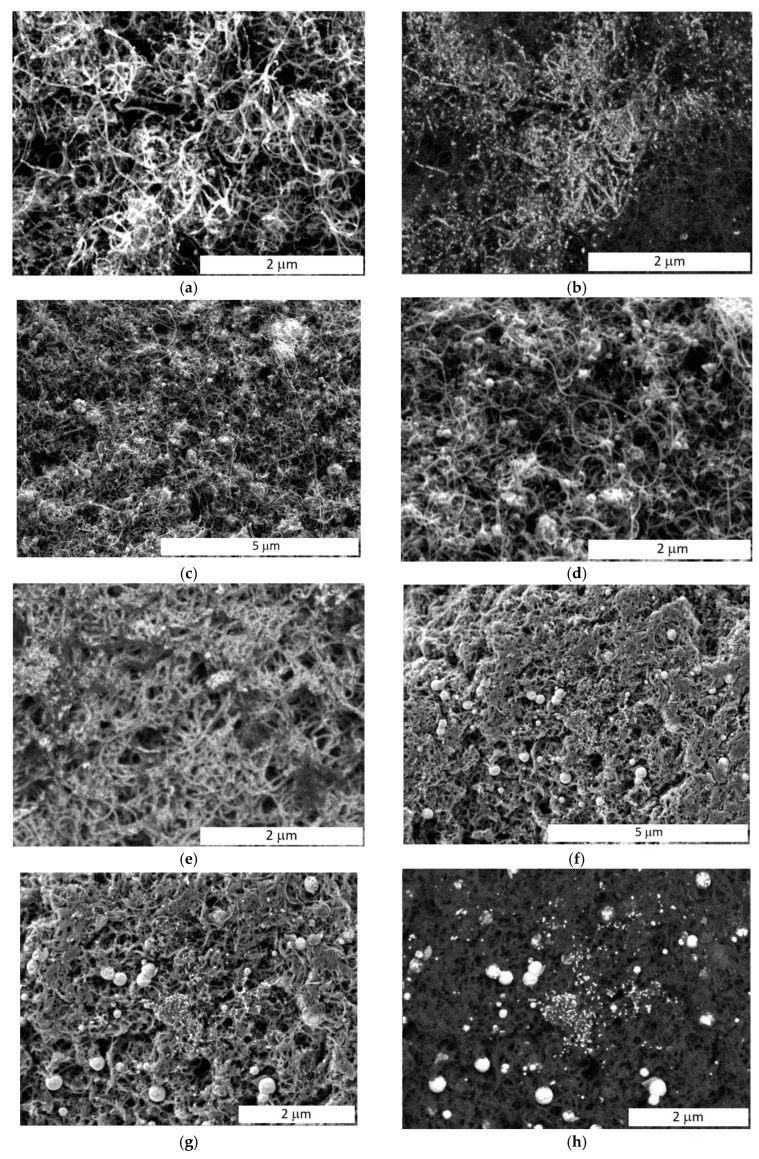
SEM images of catalysts: 60Pt/CNT_N_ (**a**,**b**), 10PtMo/CNT_N_ (**c**,**d**), 60Pt/CNT_N_ + Fumion (**e**), and 10PtMo/CNTN + Fumion (**f**–**h**) in secondary (**a**,**c**,**d**,**f**,**g**) and back-scattered (**b**,**e**,**h**) electrons.

The morphology of the obtained catalysts was studied using SEM. In the 60Pt/CNT_N_ catalyst, platinum particles up to 20 nm in size uniformly cover carbon nanotubes (Figure 3a,b). They looked like strands of beads. This is especially noticeable in SEM images obtained in backscattered electrons. On the latter, elements with a high atomic number (platinum or molybdenum) appear as lighter areas. In molybdenum-containing catalysts, the particle size distribution of heavy metals is bimodal. Along with small particles, there are larger spherical particles of up to 200 nm in size (Figure 3c,d). Unfortunately, the SEM-EDX method does not allow one to determine what elements the particles consist of, since the spot of the focused electron beam is several micrometers, while the metal particle size is tens of nanometers, i.e., in the area from which the signal is collected, there are both particles of platinum and molybdenum oxide. It can be assumed that larger particles, which are absent in samples without molybdenum, consist mainly of molybdenum oxide agglomerates. The bimodal distribution of particles containing platinum and molybdenum is especially clearly seen when comparing SEM images of identical sections of catalysts taken in secondary and backscattered electrons, for example, for the 10PtMo/CNT_N_ + Fumion catalyst (Figure 3g,h). In the 60Pt/CNT_N_ + Fumion and 10PtMo/CNT_N_ + Fumion, the introduced Fumion covers the surface of carbon nanotubes and thickens them (Figure 3f–h). This leads to denser agglomerates and a decrease in pore size, which is consistent with the BET analysis data (Table 1). However, the net of intersecting carbon nanotubes provides good electrical conductivity in all catalysts.

**Figure 4:**
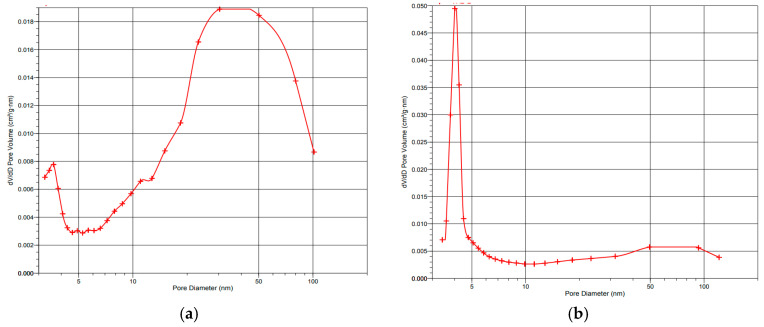
Differential mesopore size distribution based on nitrogen desorption data for 10PtMo/CNT_N_ (**a**), 10PtMo/CNT_N_ + Fumion (**b**).

Figure 4 displays the pore size distribution curves for the 10PtMo/CNT_N_ catalyst as an example, while Table 1 summarizes the porous structure characteristics of the studied systems. It is worth noting that the addition of Fumion significantly alters the porous structure of these catalysts. Although the total surface area of 10PtMo/CNT_N_ decreases by approximately 1.5-fold, the analysis of the obtained data indicates significant modifications in the microstructure of catalysts with the Fumion addition. It is noteworthy that following the introduction of Fumion the electrochemically active surface of platinum in both systems varies insignificantly (Table 2), indicating that the surface of platinum remains unblocked by the ionomer. The morphology of the catalysts, which affects reagent transport, can significantly influence the activity of the catalysts as a part of AEMFC MEA. In this regard, a catalyst with a more developed external surface provides certain advantages.

### 3.1. Electrochemical Characteristics of Catalysts under Model Conditions

**Figure 5:**
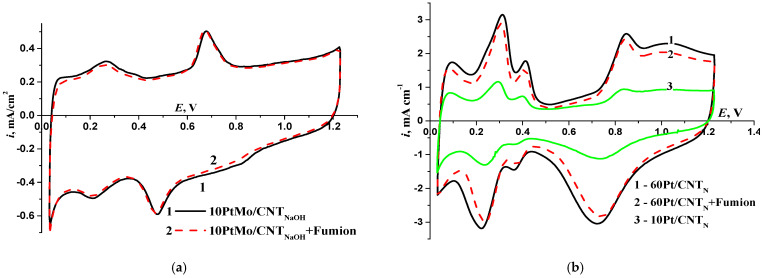
(**a**) I–V curve for 10PtMo/CNT_N_ without Fumion—1, with Fumion—2; (**b**) 60Pt/CNT_N_ without Fumion—1, with Fumion—2 and 10Pt/CNT_N_ without Fumion—3; 0.1 M KOH, 0.05 mV/s, m_cat_ = 0.15 mg cm^−2^.

To evaluate the state of the S_EAS_, I–V curves were recorded (Figure 5). The S_EAS_ of Pt for 10PtMo/CNT_N_ amounted to approximately 25 m^2^/g. Moreover, the I–V curves exhibited a redox peak at 0.7 V in the anodic reaction and 0.5 V in the cathodic reaction, which can be attributed to the molybdenum redox transformations. The current–voltage curve for the monoplatinum catalyst with and without Fumion shows a typical curve for platinum. The surface area calculated from the hydrogen adsorption/desorption area is approximately m^2^/g: 60 for 60Pt/CNT_N_ and 52 for 10Pt/CNT_N_. 

The addition of Fumion in an amount of I/C = 1/1 (where I is the mass of the ionomer and C is the mass of the carbon carrier in the catalyst) has an insignificant effect on the shape of the I–V curve, as shown in Figure 5.

**Figure 6:**
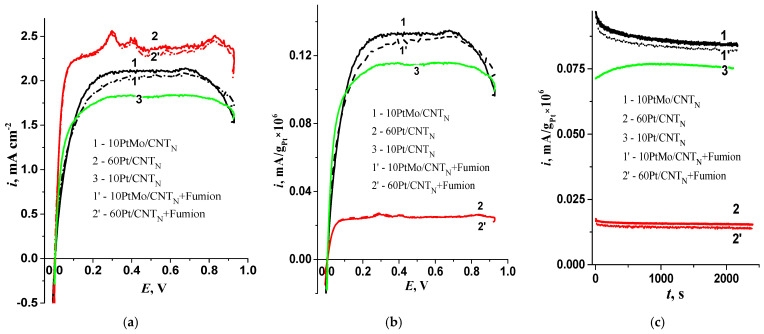
(**a**) Polarization curves for hydrogen oxidation at 1500 rpm; (**b**) Polarization curves relative to Pt mass at 1500 rpm; (**c**) Current transients at 0.4 V, 650 rpm for 10PtMo/CNT_N_—1; 60Pt/CNT_N_—2; 10Pt/CNT_N_—3 (indicated in figure), without Fumion—1, 2, with Fumion—1′, 2′; 0.1 M KOH. 0.005 V/s; m_cat_ = 0.15 mg cm^−2^.

To determine the activity of the studied catalysts, measurements were carried out under model conditions using a rotating disk electrode with and without a catalyst containing Fumion in HOR in a three-electrode cell with a liquid electrolyte. Polarization curves were recorded (Figure 6a). The 10PtMo/CNT_N_ catalyst exhibits a lower current density (*i*) compared to that of 60Pt/CNT_N_, but it is higher than *i* at 10Pt/CNT_N_ (Figure 6 curve 3) [32]). When calculating the activity per mass of platinum in the catalyst composition (Figure 6b), 10PtMo/CNT_N_ is characterized by higher values of current densities compared to monoplatinum catalysts, which confirms the synergistic effect of transition metal and platinum in the HOR.

Figure 6c demonstrates that the investigated catalysts exhibit only a slight decline in current density over time, indicating their high stability and weak influence of the added Fumion. The addition of Fumion at a ratio of I/C = 1/1 leads to a slight reduction in the electrochemical characteristics of 10PtMo/CNT_N_, while the monoplatinum catalysts remains virtually unaffected. Therefore, the addition of Fumion only has a minor effect on the electrochemical characteristics of the catalysts, as observed in the study under model conditions.

### 3.2. The Impact of Temperature on Hydrogen Electrooxidation

**Figure 7:**
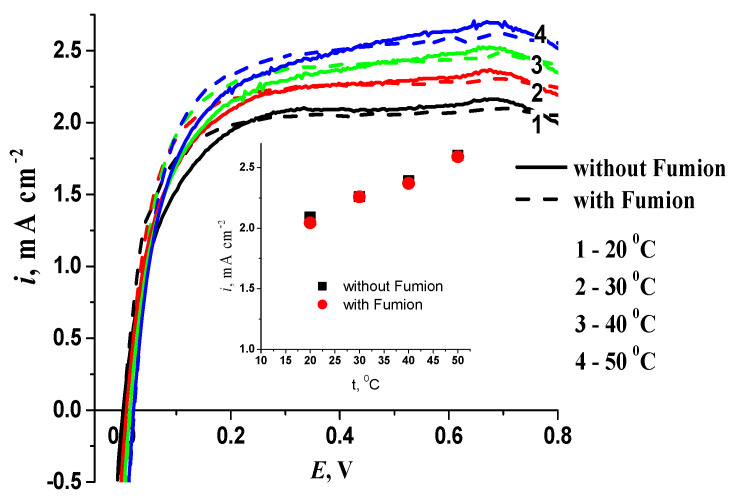
Polarization curves for hydrogen oxidation on 10PtMo/CNT_N_ at different temperatures (indicated in figure), 0.1 M KOH, 1500 rpm. The inset shows the dependence of the current density at 0.4 V on the electrolyte temperature, 0.1 M KOH, 1500 rpm.

The impact of temperature on hydrogen electrooxidation was investigated in the range of temperatures achievable by AEMFC (Figure 7).

As observed, the limiting diffusion current increases with an increase in the temperature due to an increase in the diffusion coefficient of hydrogen [32]. The addition of Fumion results in a negligible decrease in the limiting current, with a linear pattern observed regardless of the presence of the ionomer. A comparable increase in the limiting diffusion current with rising temperature is also observed for monoplatinum catalysts.

### 3.3. Optimization of MEA

The studies aimed at optimizing the MEA architecture involved evaluating the impact of various factors, such as membrane type, suspension composition (including the nature of the solvent) for forming the AL, ionomer–to–carbon (I/C) ratio and GDL type.

**Figure 8:**
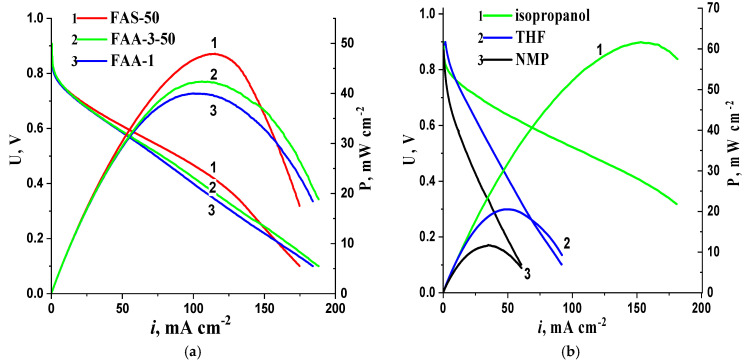
I–V curves and power density as a function of current density for AEMFC MEAs using different Fumasep membranes (**a**) and different solvents (**b**) for MEA formation. Cathode—CNTN 1 mg cm^−2^; anode—10PtMo/CNT_N_ 0.2 mg_Pt_ cm^−2^.

As shown in Figure 8a, MEAs with different Fumasep membranes exhibit similar characteristics, with a slight advantage observed for MEAs with a FAS membrane. Thus, FAS and FAA-3-50 membranes were selected for subsequent experiments. Figure 8b shows the data characterizing the effect of the solvent used for preparing the suspension for AL deposition on the AEMFC MEA parameters. The results indicate that MEA power density is highest when using the isopropanol solution. In contrast, the low performance of the MEA when using NMP-based inks, as reported in a previous study [24], could be attributed to a decrease in the number of small mesopores in the AL. In subsequent experiments, the inks were deposited using an isopropanol-based suspension.

**Figure 9:**
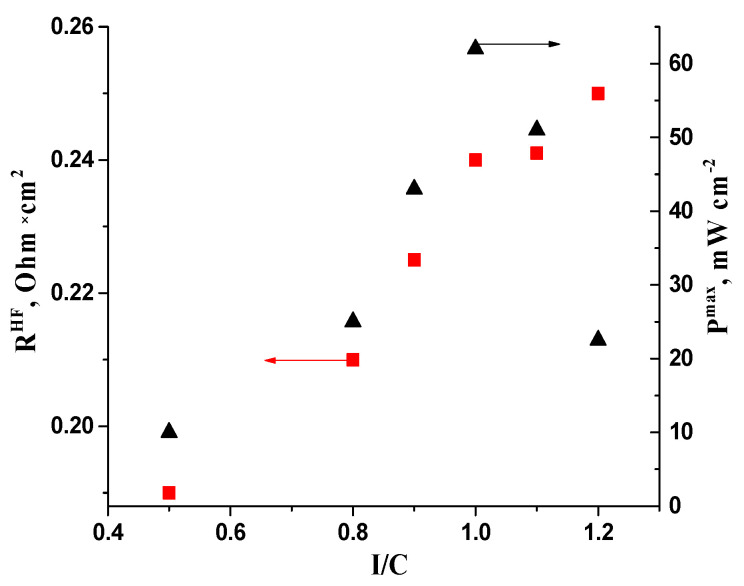
Dependencies of high-frequency resistance (▪) and maximum power density (▴) of AEMFC MEAs on I/C ratio. Cathode—CNTN 1 mg cm^−2^; anode—10PtMo/CNT_N_ 0.2 mg_Pt_ cm^−2^.

An important parameter in optimizing the AL structure is the ionomer–to–carbon carrier ratio. The introduction of ionomer in AL allows the channels for ion transport to be formed, with the minimum necessary ionomer content determined by the percolation threshold. On the basis of the I–V curves and power characteristics of MEAs shown in Figure 9, the optimal I/C = 1/1 ratio approximately corresponds to the results published in the literature [22], which describes the effect of FAA ionomer content in AL on MEA parameters with a platinum-containing cathode and anode. If the ionomer content is too low, catalyst efficiency may decrease due to the lower concentration of OH groups required in Reaction (1) within the AL volume. On the other hand, the high ionomer content (I/C = 1.2/1) presumably leads to the partial blocking of pores in AL, hindering the transport of gases and water in the reaction zone. The external surface area of 10PtMo/CNT_N_ + Fumion are twice as high as those of the monoplatinum catalyst (Table 2). The accessibility of the external surface in the delivery of gases and electrolyte is higher than that of small pores.

In contrast to the findings in the literature [22], our study showed a direct correlation between the ionomer content and the MEA resistance (Figure 9). However, it should be noted that in the literature [22], the resistance of the MEA was calculated based on the total voltage losses, excluding those which were electrochemical. This approach could lead to the overestimation of the resistance value, which was 20–30 times higher than the R^HF^ values determined in our study by the impedance method. Based on the R^HF^ parameters and current densities achieved in the AEMFC tests, it was found that the voltage drop due to ohmic losses is less than 70 mV. Therefore, electrochemical and transport losses primarily determine the overall voltage drop during MEA operation. This may explain the absence of correlation between the power characteristics and resistance of the MEA with the I/C ratio.

**Figure 10:**
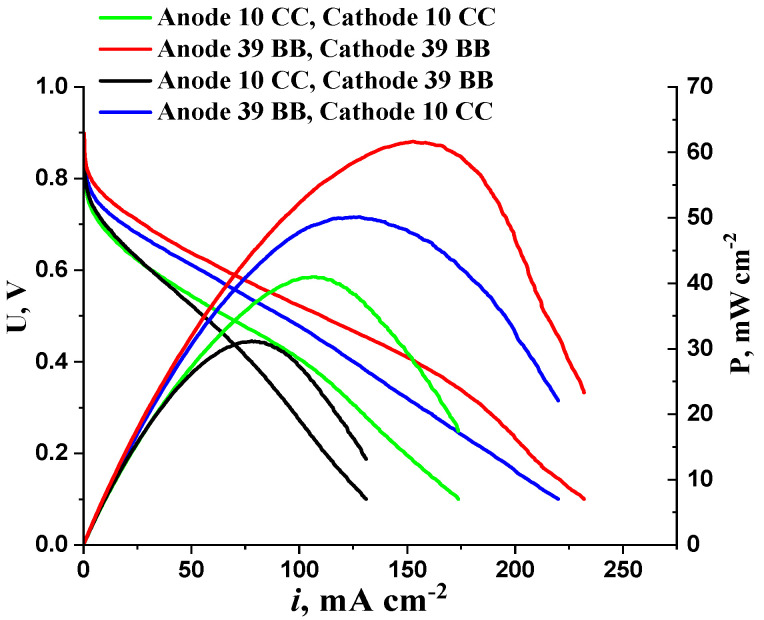
I–V curves and power density as a function of current density for AEMFC MEAs when using different GDSs for anode and cathode. Cathode—CNT_N_ 1 mg cm^−2^; anode—10PtMo/CNT_N_ 0.2 mg_Pt_ cm^−2^.

The selection of GDL for anode and cathode deposition plays a crucial role in determining the conditions of water and reagent transport in the electrochemical reaction zone. Given the results presented in Figure 10, AEMFC MEAs based on GDL (Sigracet 39 BB) exhibit better characteristics than those based on GDL 10 CC, which has a greater thickness and fluoroplastic content. However, the combination of the anode 39 BB–cathode 10 CC provides a higher power density than the combination of the anode 10 CC–cathode 39 BB. These results suggest that increasing the thickness and hydrophobic properties of the anode significantly contributes to the reduction of AEMFC MEA performance. In a recently published work, the hydrophilic–hydrophobic properties of HDS were studied and their influence on the characteristics of the MEA was shown. However, since the article refers to the influence of HDS properties on electrochemical characteristics when using proton exchange membranes, we do not discuss it in more detail [33]. Evidently, in the case of the anode deposited on the GDL 10 CC, flooding of the AL pores with water formed during hydrogen oxidation (Equation (1)) occurs. An increase in the MEA performance when passing from the combination of the anode 39 BB–cathode 10 CC to the combination of the anode 39 BB–cathode 39 BB is due to a decrease in the ORR diffusion limitations when using a thinner GDL on the cathode. On the other hand, replacing GDL 10 CC with 39 BB at the cathode has a lesser effect on MEA performance than a similar replacement at the anode. Thus, in the case of the AEMFC MEA architecture under study, the HOR voltage losses can exceed the ORR voltage losses. This circumstance makes it difficult to separate the MEA overvoltage into components for a more detailed analysis of the effect of diffusion limitations.

**Figure 11:**
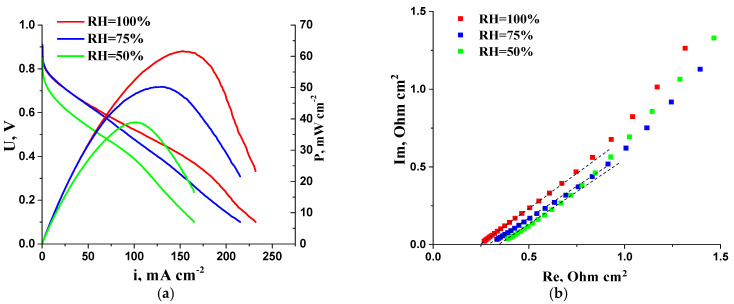
(**a**) I–V curves and dependence of power density on current density of AEMFC MEA at different RH of supplied gases; (**b**) Impedance plots of AEMFC MEA in high-frequency region at different RH of supplied gases. Cathode—CNT_N_ 1 mg cm^−2^; anode—10PtMo/CNT_N_ 0.2 mg_Pt_ cm^−2^.

Although in most studies, AEMFC MEAs were tested at humidity levels close to 100%, a decrease in hydrogen humidification from 100% to 75% was shown to increase power characteristics by reducing the flooding effect of the anode AL, according to a previous study [30]. In this work, the AEMFC was tested at different RH values to assess the effect of humidity on its performance (Figure 11). The results show that decreasing RH from 100% to 50% leads to a ~1.5-fold decrease in the maximum MEA power density. However, the R^HF^ parameter is less affected by humidity (Figure 11b), indicating that under the selected test conditions, ohmic losses due to membrane and MEA resistance have no significant influence on the current–voltage characteristics of the AEMFC.

**Figure 12:**
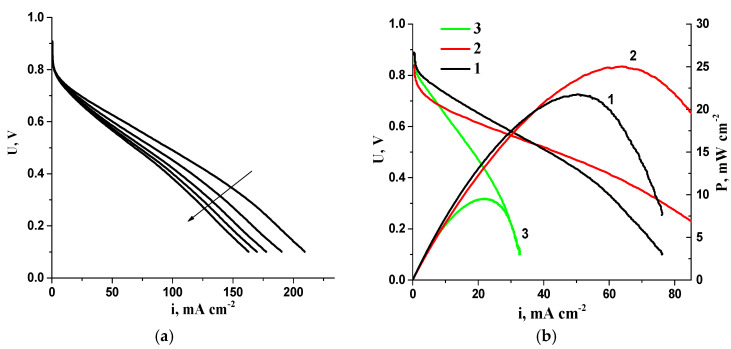
(**a**) Typical consecutive I–V curves of AEMFC MEAs with Fumasep membranes. The arrow shows the characteristic direction during cycling; (**b**) I–V curves and dependence of power density on current density for different AEMFC MEAs: 1—initial MEA; 2—MEA based on previously tested membrane and fresh electrodes; 3—MEA based on previously tested electrodes and fresh membrane. I/C = 1.2/1.

One observed negative effect during the consecutive I–V curve measurements of the AEMFC MEAs with the studied polymer electrolyte involves a rapid decrease in the characteristics (Figure 12a). In order to assess the reversibility of this effect, the MEA was disassembled following the tests, and the membrane and electrodes were subsequently re-immersed in a solution of 1 M KOH. The aged electrodes were connected to a “fresh” membrane (untested as a part of the MEA) and tested in the FC. The previously tested membrane was connected to new electrodes and tested in a separate experiment. As shown in Figure 12b, the MEA based on the previously tested membrane and fresh electrodes performed as well as the initial MEA. However, the MEA based on the previously tested electrodes and the fresh membrane performed significantly worse than the initial MEA.

To assess the practical significance of the studies, the obtained results were compared with the performance of AEMFC MEAs using a commercial 60Pt/C (HiSPEC 9100) or 60Pt/CNT_N_ catalyst for the anode and a CNT_N_-based cathode, as well as with the published literature on AEMFC MEA tests with Fumasep membranes (Table 3).

The MEA with the 10PtMo/CNT_N_ anode catalyst demonstrated superior performance compared to MEAs using catalysts containing 60Pt, as shown in Table 3. This may be due to the lower adsorption energy of the ionomer on the surface of the binary catalyst compared to that of the monoplatinum catalyst, as reported in [37], which contributes to an increase in the power density of the AEMFC MEA. In [22], a maximum AEMFC power density of 80–90 mW cm^−2^ was achieved with 0.5 mg cm^−2^ of platinum loading on both the cathode and anode, while in this study, up to 62 mW cm^−2^ was achieved using a non-platinum cathode. In [23], the maximum power density reached 41 mW cm^−2^ at 40 °C with a platinum-containing cathode and anode. In [24,34], although the characteristics of 300–400 mW cm^−2^ were obtained, the platinum loading on the cathode and anode was 0.4–0.5 mg cm^−2^, and the tests were carried out at temperatures ranging from 60 to 80 °C with thin FAA-3 membranes (20 μm). The latter factor may be significant since the transport of water from the anode to the cathode is facilitated in the case of thin membranes [38].

The data obtained show that even during short-term MEA AEMFC tests, degradation occurs, apparently due to the interaction of catalysts with the Fumion polymer electrolyte in the active layers. This degradation is irreversible, since the MEA parameters are not restored upon repeated exposure of the electrodes to the alkali solution.

## 4. Conclusions

The present study focuses on developing and examining catalysts with reduced platinum content for hydrogen–air fuel cell electrodes using the alkaline polymer membranes Fumatech. 10PtMo/CNT_N_ was used as the anode catalyst, which showed similar performance in the hydrogen oxidation reaction under model conditions as that of monoplatinum with 60 platinum content, such as 60Pt/C (HiSPEC 9100) and 60Pt/CNT_N_ [28], and higher activity compared with 10Pt/CNT_N_.

The work addressed the impact of the formation and pretreatment methods for electrodes based on anion-exchange membranes on the performance of AEMFC MEAs with the developed catalysts featuring reduced platinum content or without Pt. The results indicate that using isopropanol as a solvent for electrode deposition from suspension is preferable. The maximum MEA performance is achieved when electrodes and membranes are exposed to 1 M KOH solution for 4 h prior to conducting AEMFC MEA tests, which ensures the transition of the polymer electrolyte into the OH form.

In order to achieve maximum AEMFC MEA performance, the following conditions were optimized: MEA compression ratio of <10%, hydrogen and oxygen relative humidity of ~100% and the use of GDL (Sigracet 39 BB). The optimal ionomer–to–carbon carrier ratio in the active layers of the electrodes is 1/1.

The optimized MEA with a CNT_N_ cathode catalyst and a 10PtMo/CNT_N_-based anode exhibited a maximum power density of 62 mW cm^−2^, which surpasses the previously reported literature data for MEAs based on cathode and anode platinum catalysts and Fumasep membranes tested under similar conditions (41 mW cm^−2^ [23]). Mechanisms and ways to improve the characteristics of the MEA by optimizing the processes of MEA formation include the composition of the suspension (ink) for applying active layers to HDS; the temperature of applying active layers and humidification of gases; optimal thickness and structure of GDS; providing efficient gas supply; the degree of MEA compression; and the mode of membrane pretreatment for its conversion to the OH form.

## Figures and Tables

**Table 1:** Structural characteristics of studied catalysts.

	Parameter	S_BET_//S_EASPt_ *, m^2^/g	Total Pore Volume, cm^3^/g	Mesopore Volume, cm^3^/g; (Diameter > 2 nm)	External Surface Area//Micropore Surface Area, m^2^/g
Catalyst					
10PtMo/CNT_N_		167//24	1.87	1.79	145.7//21.4
10PtMo/CNT_N_ + Fumion		108//22	1.00	0.68	103.9//4.3
60Pt/CNT_N_		91.1//56	1.03	0.89	80.2//10.9
60Pt/CNT_N_ + Fumion		43.6//60	0.76	0.73	41.3//4.0

* On the basis of data calculated from hydrogen region in I–V curves under model conditions.

**Table 2:** Electrochemical characteristics of studied catalysts in HOR.

Catalyst	S_EAS Pt_, m^2^/g	*i*, mA/cm^2^ at 0.4 V	*η*, mV at 1.5 mA/cm^2^
	without Fumion//with Fumion		
10PtMo/CNT_N_	24//22	2//2	100//100
60Pt/CNT_N_	56//60	2.3//2.3	25//25

**Table 3:** Characteristics of AEMFC H_2_–O_2_ MEAs based on Fumasep membranes.

Membrane/Ionomer	Cathode Catalyst, %Pt (Pt Loading, mg cm^−2^)	Anode Catalyst, %Pt (Pt Loading, mg cm^−2^)	t_cell_, °C	Backpressure, atm	Pmax, mW cm^−2^	Ref.
FAA-3-20/ Fumion	40Pt/C (0.5)	40Pt/C (0.5)	50	0.5	85	[22]
FAA-3-50/ FAA-3 ionomer	20Pt/C (0.5)	20Pt/C (0.5)	40	0	41	[23]
FAA-3-20/ Fumion	44.6Pt/C (0.5)	44.6Pt/C (0.4)	60	0	300–400	[24]
FAA-3-20/ Fumion	46.7Pt/C (0.4)	46.7Pt/C (0.4)	60	0	300	[34]
FAA-3-20/ Fumion	46.6Pt/C (0.33)	46.6Pt/C (0.33)	65	With backpressure	400–500	[35]
FAA-3-50/ FAA-3 ionomer	40Pt/C (0.5)	40Pt/C (0.5)	60	0	75–140	[25]
FAA-3-50/ Fumion	NFC@Fe/Fe_3_C (0)	40Pt/C (0.4)	60	0	96	[36]
FAA-3-50, FAS/ Fumion	CNT_N_ (0)	10PtMo/CNT_N_ (0.4)	40	0	62	This work
	CNT_N_ (0)	60Pt/CNT_N_ (0.4)	40	0	32	

## Data Availability

Data will be made available on request.
